# Martensitic Transformation and Magnetic-Field-Induced Strain in High-Entropy Magnetic Memory Alloy Ni_20_Mn_20_Ga_20_Gd_20_Co_20_ by Hot-Magnetic Drawing

**DOI:** 10.3390/ma15082785

**Published:** 2022-04-11

**Authors:** Jia Ju, Mengfei Fang, Liguo Shuai, Kang Yin

**Affiliations:** 1 Jiangsu Key Laboratory of Advanced Structural Materials and Application Technology, School of Materials Science and Engineering, Nanjing Institute of Technology, Nanjing 211167, China; fangmengfeinjit@163.com; 2 Jiangsu Key Laboratory of Hazardous Chemicals Safety and Control, College of Safety Science and Engineering, Nanjing Technology University, Nanjing 210009, China; 3 Jiangyin Electrical Alloy Limited Liability Company, Wuxi 214423, China; 4 School of Mechanical Engineering, Southeast University, Nanjing 211189, China; 5 China Medical City Institute of Biomedicine and Medical Devices, Southeast University, Taizhou 225300, China

**Keywords:** ferromagnetic-shape memory alloys, high-entropy magnetic memory alloy, Ni_20_Mn_20_Ga_20_Gd_20_Co_20_, hot-magnetic drawing, martensitic transition temperature, magnetic-field-induced strain

## Abstract

The wires with chemical composition Ni_20_Mn_20_Ga_20_Gd_20_Co_20_ were prepared by hot-magnetic drawing and the microstructure evolution characteristics, martensitic transformation and MFIS process were investigated in detail, respectively. The results showed that a multiphase structure with γ phase and martensite was observed in samples when the magnetic field was 0 T to 0.2 T during the hot-magnetic drawing process. With the magnetic field increased to 0.5 T, due to the atomic diffusion by severe thermoplastic deformation and high external magnetic field, a single-phase structure with L1_0_ type twin martensite was found in the sample. Moreover, an obvious increasing trend in martensitic transformation temperature in the sample was found by the enhancement of the magnetic field during the hot-magnetic drawing process. The highest phase transition temperature rose to about 600 °C when the magnetic field reached 0.5 T. Finally, the property of SME and MFIS in the sample can be enhanced by the magnetic field increasing during the hot-magnetic drawing process, excellent performance of SME was obtained at low total strain, and MFIS was achieved at 4.47% at a magnetic field of 8007 Oe in the sample in the 0.5 T magnetic field during the hot-magnetic drawing process.

## 1. Introduction

With the great development of intelligent and sensor technology, ferromagnetic-shape memory alloys (FSMAs) have become one of the most potentially intelligent drive materials in recent years due to their excellent characteristics [1,2,3,4], i.e., over 10% magnetic-field-induced strain (MFIS) and a quick response of less than a millisecond for single-crystal Ni_2_MnGa-based FSMAs [5,6]. In this case, many scholars have shown great interest in the research of FSMAs [7]. Unfortunately, for polycrystalline FSMAs, as the potential materials for high-frequency actuators and sensors, low martensitic transformation temperature and small MFIS always restrict the application of this kind of material [8,9,10].

In the research during the last decades, high-entropy FMSAs with high martensitic transformation temperature, which are designed by the multielement metallic alloys close to equimolar composition, have become a research hotspot [11,12,13]. For high-entropy alloys, usually in the form of a solid solution, bulk metallic glasses and even intermetallic compounds, with a high mixing entropy compared with the conventional alloy, can be ensure a high stability of the phase and excellent properties due to high solution of alloying elements and sluggish diffusion [14]. However, in the case of equimolar multielement, a large number of second phases usually appear in high-entropy FMSAs, which can apparently be a weakening of MFIS [15]. 

Generally, the shape of driving materials in actuators and sensors is a wire; by now, most research on the preparation of FSMA wire is focused on conventional deformation [16]. Among various techniques, hot drawing, which provides a larger single-pass deformation without heat treatment during the intervals, is more suitable for FSMA wire manufacturing [17]. Moreover, as one kind of efficient self-reinforcement technology, hot drawing will further promote the orientation and arrangement of grains along the direction of the out force, which is beneficial for the improvement in the grain orientation of FSMAs and MFIS of FSMA wire [18]. On the other hand, with the application of external magnetic fields, the material properties can be improved, due to accelerating the phase transformation kinetics and increasing the grain orientation of magnetic phases [19]. 

Therefore, in this study, the wire of polycrystalline high-entropy Ni_20_Mn_20_Ga_20_Gd_20_Co_20_ (processed by hot-magnetic drawing) was the subject, and its microstructure evolution during hot-magnetic drawing processes was systematically investigated. To emphasize the influence of martensitic transformation and the driving force of MFIS by different magnetic fields, the microstructure evolution characteristics, martensitic transformation and MFIS process were investigated in detail during hot-magnetic drawing.

## 2. Materials and Methods

### 2.1. Sample Preparation

**Figure 1:**
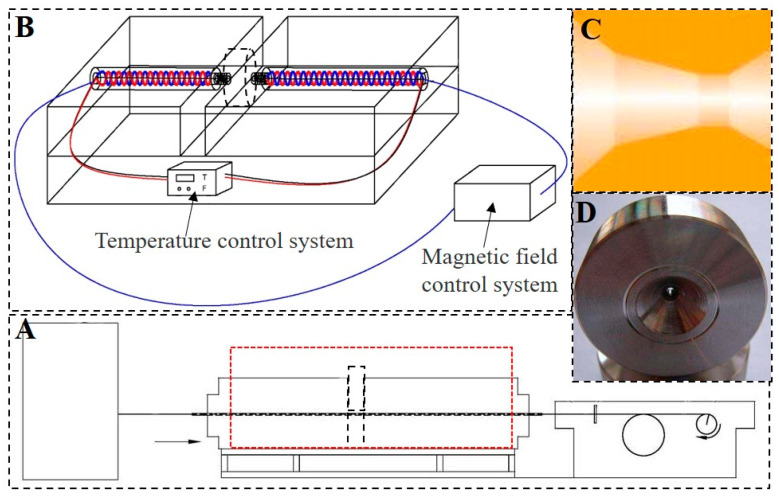
The structure of self-made hot-magnetic drawing device. (**A**): the whole structure of sample drawing equipment; (**B**): the part of hot-magnetic drawing; (**C**): section structure of drawing die; (**D**): drawing die.

The ingot with chemical composition Ni_20_Mn_20_Ga_20_Gd_20_Co_20_ (at.%) was produced using high-purity elements (more than 99.99%, provided by China New Metal Materials Tech. Co., Ltd., Beijing, China) by vacuum induction melting (VIF-50, provided by Shandong Huaxin Electric Furnace Co., Ltd., Shandong, China) and cast into 60 mm diameter cylindrical ingot. In order to ensure uniform chemical composition of the alloy, the ingot was vacuum-homogenized at 1000 °C for 72 h. Then, the ingot was extruded at 500 °C with an extrusion ratio of 16. The cylindrical sample was cut from the extruded bar with a size of 15 mm × 80 mm (diameter × height). The hot-magnetic drawing was carried out using self-made device (the structure of self-made device is shown in Figure 1) with 10 mm/s (drawing speed) and 20% single-pass deformation at 500 °C. The diameter sample was reduced from 1 mm to 0.31 mm after 11 passes of hot-magnetic drawing with 0–0.5 T magnetic field and the samples with different hot-magnetic drawing are presented in Table 1.

### 2.2. Microstructure Observation and Performance Test

Microstructure and energy dispersive spectroscopy (EDS) of samples were determined by scanning electron microscopy (SEM, FEISirion200, provided by Thermo Fisher, Hillsboro, Oregon, USA). The phase position and crystal structure of samples were analyzed by X-ray diffraction (XRD, D8 Advance X, provided by Bruker AXS, Karlsruhe, Baden-Wurttemberg, Germany) at room temperature. The martensitic transition temperature of samples was characterized by differential scanning calorimeter (DSC, STA449 F3, provided by NETZSCH Scientific Instruments Trading Ltd., Shanghai, China). The finer microstructure and crystal structure of martensite phase were characterized by transmission electron microscopy (TEM, provided by Tecnai G2 F20, FEI, Hillsboro, Oregon, USA) and selected area electron diffraction (SAED). The volume fraction of different phases was determined by means of the image analyzer. The shape memory effect (SME) of the sample was tested by pre-compressing three times to the specific strain with unloading and heating to 300 °C for every recovery. The strain recovery ratio (R_SME_) can be obtained by Equation (1) [20]:

R_SME_ = [(ΔL − L_o_ − L_r_)/ΔL] × 100% (1)

where ΔL is the length after unloading, L_o_ and L_r_ are the original length and the length after recovery, respectively. The MFIS of sample was measured via metal strain gauges with a magnetic field of 0–9000 Oe.

## 3. Results and Discussion

### 3.1. Microstructure and Composition Distribution

**Figure 2:**
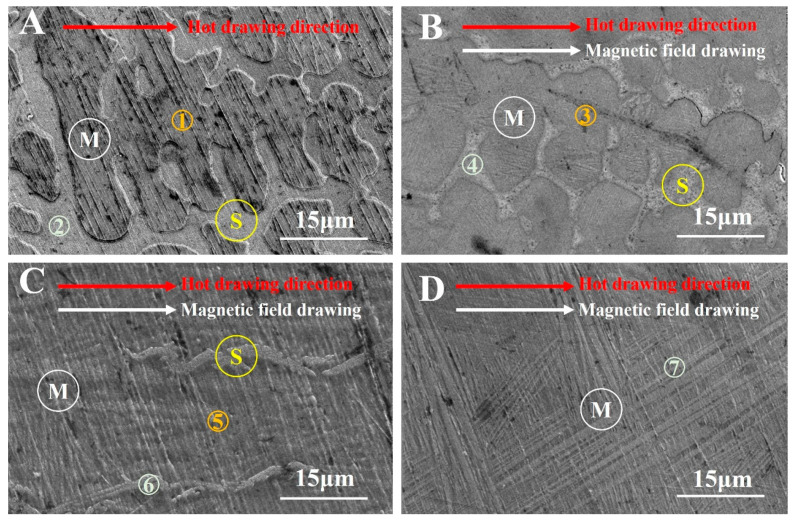
Microstructure characteristics of samples by SEM observation. (**A**) Sample a; (**B**) Sample b; (**C**) Sample c; (**D**) Sample d.

Figure 2 shows the microstructure characteristics of the sample by SEM observation. As seen in Figure 1a, the sample appeared as a typical multiphase structure, including a inclined plate strip matrix structure (Figure 2a Ⓜ) and a dendritic second phase structure distributed along the hot drawing direction (Figure 2a Ⓢ). A large number of second phases appeared at the grain boundary, indicating that high entropy designed with an equal atomic ratio of Ni-Mn-Ga-Gd-Co has serious element segregation. Seen from Figure 1b, the plate strip matrix (Figure 2b Ⓜ) and second phase (Figure 2b Ⓢ) shows an obvious elongation trend along the hot drawing direction and the magnetic field direction. In addition, the proportion of the second phase also tends to decrease significantly when applying the 0.1 T magnetic field during hot-magnetic drawing. When the magnetic field strength increased from 0.1 T to 0.2 T (Figure 2c), an obvious change of microstructure occurred; that is, the matrix phase (Figure 2c Ⓜ) gradually replaces the second phase structure. Meanwhile, the dendritic second phase (Figure 2c Ⓢ), which distributed along the hot drawing direction and the magnetic field direction, gradually changes and gathers more, forming a strip structure distributed along the hot drawing direction and the magnetic field direction. Finally, when the magnetic field reached 0.5 T, only the strip matrix phase (Figure 2d Ⓜ) can be observed in Figure 2d, indicating that the second phase has been completely replaced by the matrix phase under the dual action of strong external magnetic field and thermal drawing. 

In order to clarify the composition change in samples under the hot-magnetic drawing, EDS analysis of the samples after hot-magnetic drawing is given in Table 2. It seems clear that the alloy elements have obvious distribution changes after hot-magnetic drawing with different intensities of the external magnetic field. For the alloy after hot-magnetic drawing without a magnetic field, the element distribution of the matrix has approximately equal atomic ratio with each element (scan area ① in Figure 2a, atomic ratio: Ni:Mn:Ga:Gd:Co ≈ 1:1:1:1:1). In comparison, the element distribution in the second phase shows a phenomenon of elements (Gd and Co) serious aggregation (scan area ② in Figure 2a, atomic ratio: Ni:Mn:Ga:Gd:Co≈1:1:1: 2.4:2.6). When the hot-magnetic drawing with 0.1 T magnetic field, the element distribution of the matrix still maintains an equal atomic ratio (scan area ③ in Figure 2b, atomic ratio: Ni:Mn:Ga:Gd:Co ≈ 1:1:1:1:1). However, the second phase, which is segregated at the grain boundary, still has more obvious aggregation of Gd and Co (scan area ④ in Figure 2b, atomic ratio: Ni:Mn:Ga:Gd:Co ≈ 1:1:1:2.1:2.2). With the magnetic field increased to 0.2 T during the hot-magnetic drawing process, the matrix phase still maintains the element distribution with an equal atomic ratio (scan area ⑤ in Figure 2c, atomic ratio: Ni:Mn:Ga:Gd:Co ≈ 1:1:1:1:1), and the aggregation of Gd and Co is obviously weakening in the second phase (scan area ⑥ in Figure 2c, atomic ratio: Ni:Mn:Ga:Gd:Co ≈ 1:1:1:1.5:1.7). When the magnetic field increased to 0.5 T, with the disappearance of the second phase, the composition distribution of the retained matrix phase always maintains approximately the same atomic ratio (scan area ⑦ in Figure 2d, atomic ratio: Ni:Mn:Ga:Gd:Co ≈ 1:1:1:1:1). 

**Figure 3:**
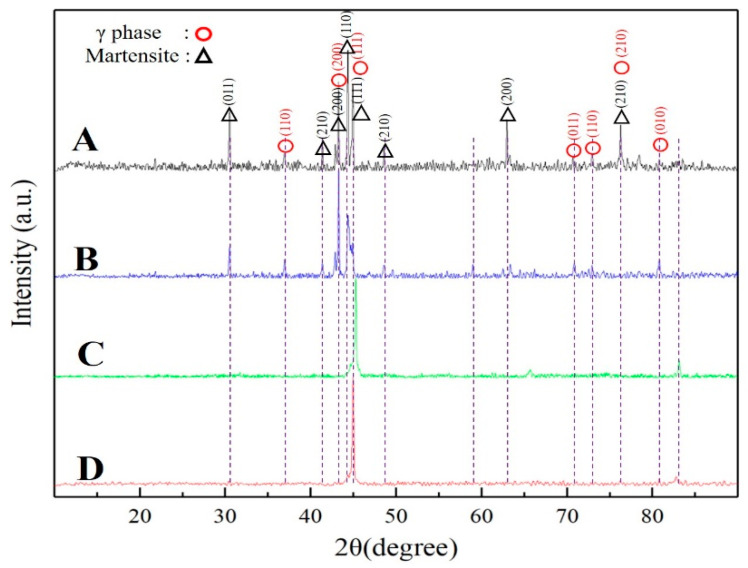
The XRD patterns of samples (A) Sample a; (B) Sample b; (C) Sample c; (D) Sample d.

The XRD patterns of samples with different hot-magnetic drawing are shown in Figure 3. Obviously, from the XRD pattern analysis, two phases were identified in samples (a, b and c): martensite phase (L1_0_ type) and γ phase (A1 type), indicates that the matrix and the second phase, which can be observed in the SEM images, are martensite phase and γ phase, respectively. For sample d, only the martensite phase diffraction peak was found, which confirmed that the sample d is a single phase (martensite phase) structure as observed in Figure 1d. The peaks, which can be indexed as L1_0_ type martensite in all samples, suggest that martensitic transformation has completely occurred at room temperature. Furthermore, from the diffraction peak intensity changes of different phases during hot-magnetic drawing, two obvious phenomena are shown in Figure 3. One is that the diffraction peak intensity of martensite phase at 45.1° becomes abnormally stronger. Another is that the diffraction peak intensity of γ phase at 36.8°, 43.9° and 83.2° becomes more weaker and finally disappears. These phenomena show that the sample undergoes the preferred orientation of crystal growth during hot-magnetic drawing.

### 3.2. Phase Transition Temperature and Martensite Structure

**Figure 4:**
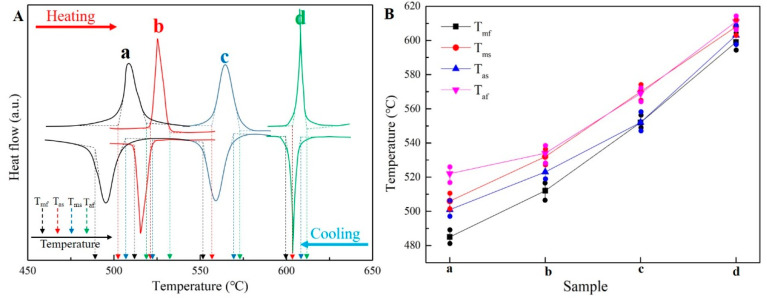
DSC curves (**A**, (a) Sample a; (b) Sample b; (c) Sample c; (d) Sample d) plotting heat flow as a function of temperature and phase transition temperature (**B**) for samples in different hot-magnetic drawing conditions.

The DSC curves and the phase transition temperature (including martensite-start (T_ms_), martensite-finish (T_mf_), austenite-start (T_as_) and austenite-finish (T_af_)) for the samples can be seen in Figure 4. In the DSC curves (Figure 4A), only one endothermic and exothermic peak can be found, indicating that a reversible one step austenite and martensite transformation has occurred during the heating and cooling process, respectively. The T_mf_ of sample (a: 485 °C, b: 512 °C, c: 552 °C and d: 599 °C) is much higher than room temperature, meaning that the matrix is martensitic at room temperature, which is consistent with the observation results of the microstructure. Furthermore, during the cooling and heating cycle process, the martensite transition temperature (T_ms_ and T_mf_) and austenite transition temperature (T_as_ and T_af_) have an obvious rising trend with the increase inmagnetic field strength in hot-magnetic drawing (as shown in Figure 4B). For the sample without a magnetic field, the phase transformation temperature is around 500 °C (T_ms_ = 506 °C, T_mf_ = 485 °C; T_as_ = 501°C, T_af_ = 522°C). During the hot-magnetic drawing with 0.1 T magnetic field, the phase transformation temperature of the sample rose by about 12–27 °C (T_ms_ = 532 °C, T_mf_ = 512 °C; T_as_ = 523 °C, T_af_ = 534°C). When the magnetic field rises to 0.2 T during hot-magnetic drawing, the phase transition temperature further increases by about 29–39 °C (T_ms_ = 570 °C, T_mf_ = 551 °C; T_as_ = 552 °C, T_af_ = 569 °C). Finally, when the magnetic field reaches 0.5 T, the phase transition temperature rises to about 600 °C (T_ms_ = 608 °C, T_mf_ = 599 °C; T_as_ = 603 °C, T_af_ = 611 °C). 

**Figure 5:**
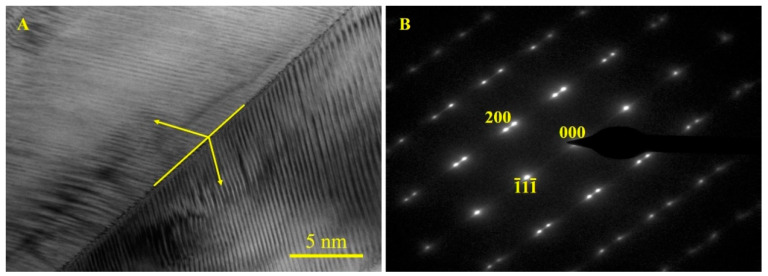
The TEM image (**A**) and SAED analysis (**B**) of martensite.

In order to observe the detailed morphology of martensite and determine the structure, the TEM and SAED analysis of sample d were carried out and the results are shown in Figure 5. From Figure 5A, the martensite morphology of the sample obviously shows typical twin martensite characteristics, which consist of uniform parallel black and white stripes alternatively at approximately a 45° incline angle. On the other hand, from the SAED analysis in Figure 5B, the diffraction spots along (001) zone axis suggest that the structure of martensite is the L1_0_ twin structure with a ($\bar{1}$1$\bar{1}$) twinning plane.

### 3.3. Shape Memory Effect and Magnetic-Field-Induced Strain

**Figure 6:**
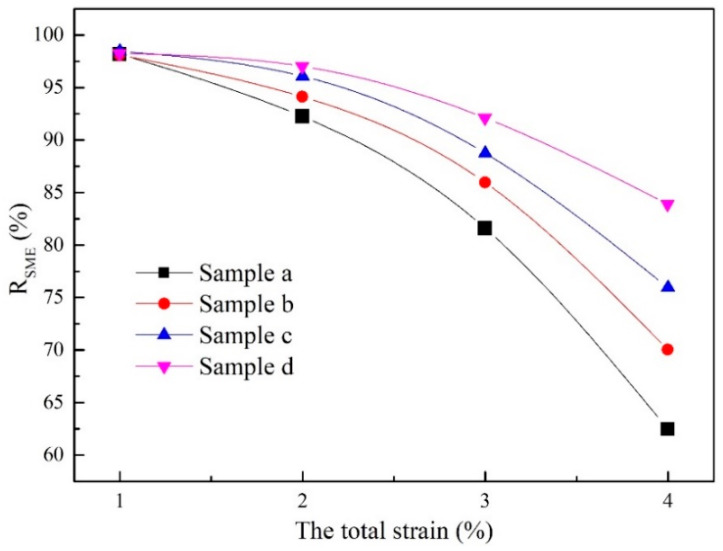
The R_SME_ curves of samples with different total strain.

The R_SME_ curves of samples with different total strain (ε, engineering strain, which is obtained by ε = (L − L_0_)/L_0_. where L and L_0_ are original gauge length of the sample and length of the sample after deformation, respectively) are presented in Figure 6. Clearly from the curves, the higher magnetic field during the hot-magnetic drawing process, the more stable shape the memory effect of the sample, due to the gentler slope of the RSME curve. At 1% total strain, the samples almost completely restored to the original shape (sample a: R_SME_ = 98.2%; sample b: R_SME_ = 98.9%; sample c: R_SME_ = 99.2%; sample d: R_SME_ = 99.1%). When the total strain rises to 2%, the strain recovery ratio shows a slight downward trend of varying degrees; the R_SME_ values of different samples was still close (such as sample a: R_SME_ = 92.3%; sample b: R_SME_ = 95.6%; sample c: R_SME_ = 96.1%; sample d: R_SME_ = 96.9%). The higher R_SME_ values (>90%) show that the sample has an excellent shape memory effect at lower total strain (≤2%). However, with the total strain further increasing (3%), the R_SME_ values decrease significantly, and a great difference appears between sample a-d (sample a: R_SME_ = 81.6%; sample b: R_SME_ = 86.7%; sample c: R_SME_ = 89.4%; sample d: R_SME_ = 93.2%). When the total strain reaches 4%, the R_SME_ values decrease further (sample a: R_SME_ = 62.3%; sample b: R_SME_ = 68.9%; sample c: R_SME_ = 75.6%; sample d: R_SME_ = 84.3%). The samples (sample a and b), with the R_SME_ values (<70%) at the higher total strain (≥3%), indicate that the samples a and b cannot maintain a good shape memory effect at higher total strain.

**Figure 7:**
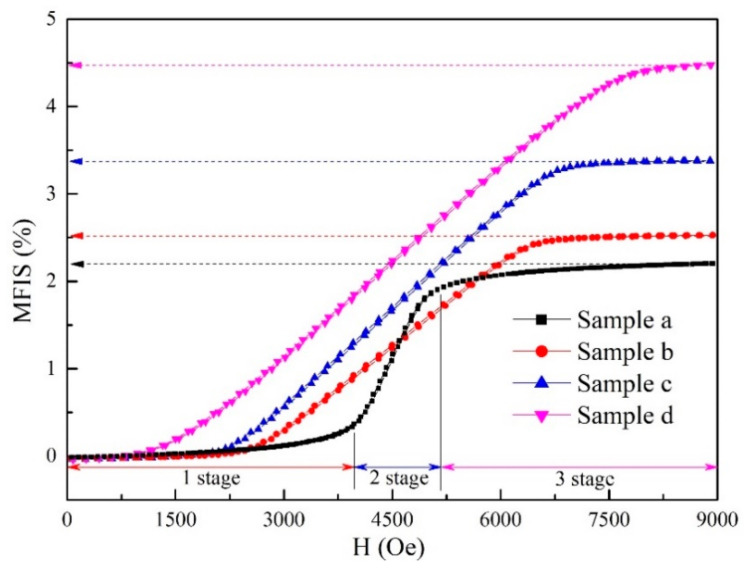
The MFIS curves of samples with different applied magnetic fields.

The MFIS curves of samples with different applied magnetic fields are exhibited in Figure 7. As presented in the MFIS curves, the samples with magnetic field during the hot-magnetic drawing process show an apparent MFIS at a magnetic field of 0–9000 Oe. The maximum MFIS of sample a (without magnetic field during the hot-magnetic drawing process) is 2.13% at a magnetic field of 7326 Oe. The sample b, which was prepared at 0.1 T during the hot-magnetic drawing process, reached the maximum MFIS (2.47%) at a magnetic field of 6590 Oe. The maximum MFIS continues to rise to 3.32% at a magnetic field of 7338 Oe in sample c (0.2 T during the hot-magnetic drawing process). Finally, sample d, with the 0.5 T during the hot-magnetic drawing process, achieved the maximum MFIS (4.47%) at a magnetic field of 8007 Oe. 

On the other hand, as shown in Figure 7, the MFIS curves are S-shaped and divided into three stages:

No. 1 stage: mainly distributed in the region with a low magnetic field. At this time, there is almost no MFIS in the sample;

No. 2 stage: mainly distributed in the region with a medium magnetic field. At this time, the sample obtains obvious MFIS;

No. 3 stage: mainly distributed in the region with a high magnetic field. At this time, the sample obtains the maximum MFIS.

### 3.4. Discussion

#### 3.4.1. Discussion on Microstructure and Martensite Transition

**Figure 8:**
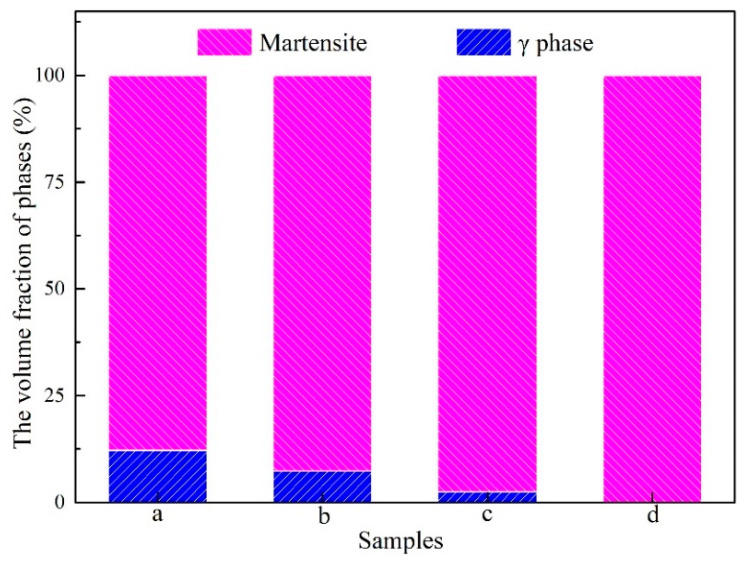
The volume fraction of γ phase and martensite in the samples.

In the research on the microstructure, two obvious microstructure changes were in the samples: 

(1) Decreasing volume fraction of the γ phase. Figure 8 exhibits the volume fraction of γ phase and martensite in the samples. The volume fraction of γ phase shows obvious decreasing from 12.17% to 0% (sample a: 12.17%; sample b: 7.32%; sample c: 2.51%; sample d: 0%). The reason is mainly attributing this change to the high-entropy composition design of the sample with equal atomic ratio and the directional diffusion of atoms during the hot-magnetic drawing process. As is well known, a multi-phase structure, caused by equimolar multielement composition, is one of the microstructural characteristics of high-entropy alloy [21]. In this case, in the sample with chemical composition Ni_20_Mn_20_Ga_20_Gd_20_Co_20_ (at.%) in this study, the aggregation of elements Gd and Co at the grain boundary occurred and γ phase formed. On the other hand, during hot-magnetic drawing, the atomic diffusion in the sample is intensified, owing to severe thermoplastic deformation and external magnetic field in the drawing direction. Therefore, the segregation of the elements Gd and Co in the sample is weakened continuously and results in the volume fraction of γ phase decreasing continuously during hot-magnetic drawing.

(2) The preferential direction of crystal. In previous studies [22,23,24], when the external magnetic field was applied during the solidification and deformation of the material, the grains of the material rotated with the external magnetic field and the deformation direction, resulting in the phenomenon of preferential growth. In this study, from XRD analysis, during hot-magnetic drawing, with the substitution of martensitic phase for the γ phase, the martensitic phase shows a strong crystal-preferred orientation in (111) due to the superposition effect of thermal drawing and the external magnetic field.

In previous research on martensite transition [25,26,27], the martensitic transformation temperature for FSMAs is very sensitive to the composition of the material, owing to a directly proportional relationship between the valence electron concentration (R_e/a_) and the martensitic transformation temperature in the material_._ The function relation between R_e/a_ and T_ms_ in FMSAs can be expressed in Equation (2) [28,29]:

T_ms_/10000 = −1.478 + (0.1933 × R_e/a_) − 0.1643 × (S^↑^ − S^↓^); R_e/a_ = ∑(V_i_ × P_i_) (2)

where S^↑^ and S^↓^ are the spin-up electrons and spin-down electrons of atomic; V_i_ and P_i_ are the atomic valence electron and the proportion of element i, respectively. In this study, the diffusion of element Gd and Co, which segregated in the γ phase, increased with the intensity of external magnetic field increasing from 0.1–0.5 T during hot-magnetic drawing. Thus, the R_e/a_ values of the sample rose continuously and resulted in a relative position change of the Brillouin zone boundary and Fermi surfaces. This eventually led to an obvious increasing of martensitic transformation temperature in the study sample.

#### 3.4.2. Discussion on SME and MFIS

For FSMAs, both good SMF and large MFIS depend on two conditions: twin martensite and the driving force during rearrangement of twin martensite [30]. According to the results of microstructure analysis, as the external magnetic field increases from 0–0.5 T during hot-magnetic drawing, the martensite in the sample gradually replaces the γ phase, which can provide the microstructure (twin martensite) condition for SMF and MFIS. On the other hand, the driving force, as a main condition for SMF and MFIS, can be expressed by dimensionless field parameter (D_fp_) using Equation (3) [31].

D_fp_ = Z_e_/2 K_u_; Z_e_ = M_s_·H (3)

where Z_e_, K_u_, M_s_ and H are the Zeeman energy, the magnetocrystalline anisotropy energy, the saturation magnetization and the applied magnetic field of the sample, respectively. 

**Figure 9:**
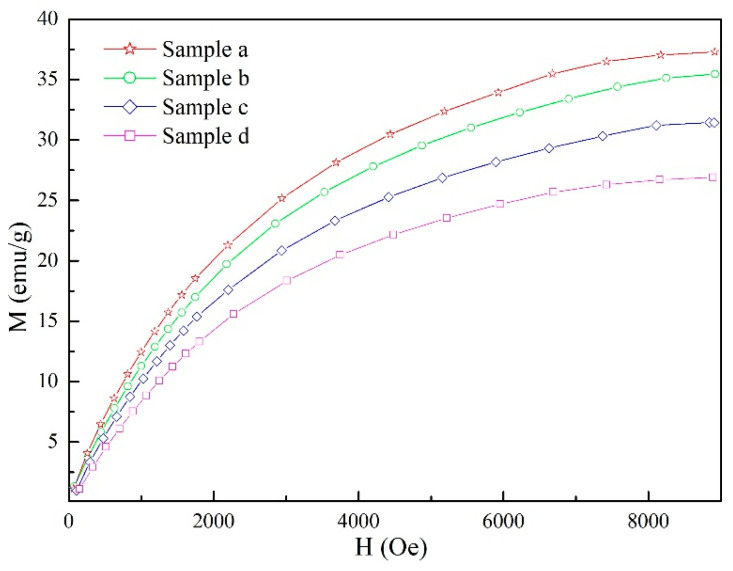
The magnetization curves of study samples.

During the rearrangement of twin martensite, the resistance and impetus of the twin martensite rearrangement are supplied by the Z_e_ and K_u_, respectively [31]. Therefore, this means that impetus is greater than resistance if the D_fp_ value is less than 1 and a MFIS occurs because of the twin boundaries in variants of the twin martensite rearrangement. As a key parameter of driving force, M_s_ is analyzed by the magnetization curves of the study sample in Figure 9. Obviously, with the external magnetic field rising from 0.1–0.5 T during hot-magnetic drawing, the M_s_ value of samples exhibits a decreasing trend (sample a: 37.2384 emu/g; sample b: 34.6394 emu/g; sample c: 28.6675 emu/g; sample d: 24.9637 emu/g). According to previous studies, the resistance of twin martensite remigration can be weakened by the decreasing of M_s_.

**Figure 10:**
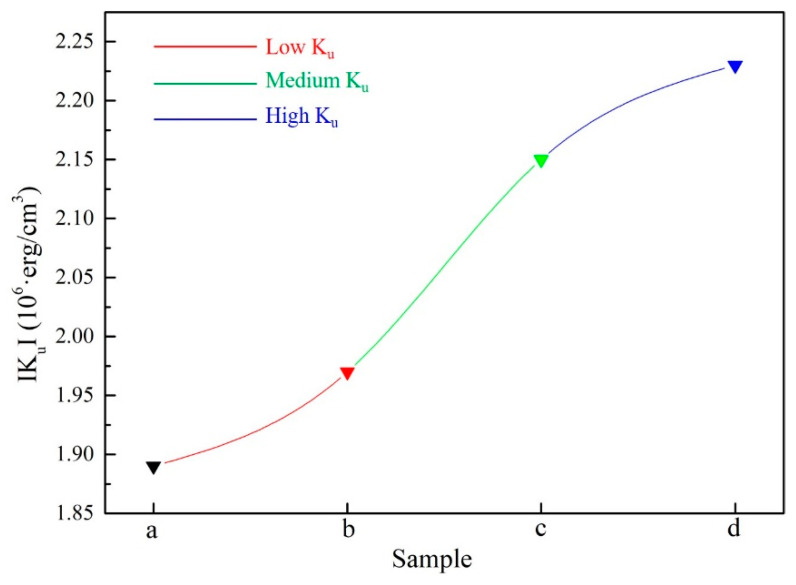
The Ku value of study alloys.

Another key parameter of driving force, along with the impetus of the twin martensite rearrangement, K_u_ is calculated using the Sucksmith–Thompson method in Equation (4) and the result is shown in Figure 10 [31].

2 × (K’_2_/M^2^_s_) + 4 × (K’_4_/M^4^_s_) × M^2^ = H_e_/M; K_u_ ≈ K’_2_ + K’_4_ (4)

therein, K_u_ is the uniaxial magnetocrystalline anisotropy constant; K’_2_ and K’_4_ are the second- and fourth-order magnetocrystalline anisotropy constants; M and H_e_ are the magnetization and applied field, respectively. From Figure 10, the K_u_ value exhibits an obvious increasing trend as the external magnetic field rises during hot-magnetic drawing (sample a: 1.89 × 10^6^·erg/cm^3^; sample b: 1.97 × 10^6^·erg/cm^3^; sample c: 2.15 × 10^6^·erg/cm^3^; sample d: 2.23 × 10^6^·erg/cm^3^) and results in the impetus of the twin martensite remigration enhanced during this process. 

Based on the driving force analysis, the three stages of MFIS curves can be explained as follows:

No. 1 stage: with low magnetic field, the driving force is too low to migrate the twin martensite and no MFIS is obtained in the sample;

No. 2 stage: with medium magnetic field, the driving force apparently increases as the applied magnetic field increases and reaches the threshold of twin martensite migration, resulting in the twin martensite rearrangement.

No. 3 stage: with high magnetic field, the magnetization and the driving force of the sample reach the saturation value and the twin martensite variants gradually complete the rearrangement, which is caused by the MFIS of the sample reaching the maximum value.

## 4. Conclusions

In this work, the wires with chemical composition Ni_20_Mn_20_Ga_20_Gd_20_Co_20_ were prepared by hot-magnetic drawing. The microstructure evolution characteristics, martensitic transformation and MFIS process were investigated in detail and we drew the following conclusions:

(1) A multiphase structure (γ phase and martensite, which was distributed along both the hot drawing direction and magnetic field direction) was observed in samples a, b and c when the magnetic field was 0 T, 0.1 T and 0.2 T during the hot-magnetic drawing process. When the magnetic field increased to 0.5 T, a single-phase structure (martensite) was found in the sample d, due to the atomic diffusion by severe thermoplastic deformation and high external magnetic field.

(2) An obvious increasing trend in martensitic transformation temperature in the sample was found by the enhancement of the magnetic field during the hot-magnetic drawing process. The highest phase transition temperature rose to about 600 °C when the magnetic field reached 0.5 T, and this established the possibility of high temperature application of high-entropy FSMAs for high-frequency actuators and sensors. 

(3) The martensite structure of the sample, with the hot-magnetic drawing process, exhibited typical twin martensite characteristics in L1_0_ type and formed twin grains in the ($\bar{1}$1$\bar{1}$) twinning plane. 

(4) The property of SME and MFIS in the sample can be enhanced by the magnetic field increasing during the hot-magnetic drawing process due to twin martensite evolution and the driving force enhancement. Finally, the excellent performance of SME was obtained at low total strain and MFIS was achieved in 4.47% at a magnetic field of 8007 Oe in the sample with the 0.5 T magnetic field during the hot-magnetic drawing process. Therefore, to realize FSMAs with high SME and MFIS, more preferential growth of martensite should be controlled, and the driving force of the twin martensite remigration should be enhanced further.

## Figures and Tables

**Table 1:** The different hot-magnetic drawing conditions of samples.

Samples	Nominal Compositions (at.%)	Hot-Magnetic Drawing Condition			
		Temperature	Magnetic Field	Drawing Speed	Single-Pass Deformation
a	Ni_20_Mn_20_Ga_20_Gd_20_Co_20_	500 °C	0 T	10 mm/s	20%
b	Ni_20_Mn_20_Ga_20_Gd_20_Co_20_	500 °C	0.1 T	10 mm/s	20%
c	Ni_20_Mn_20_Ga_20_Gd_20_Co_20_	500 °C	0.2 T	10 mm/s	20%
d	Ni_20_Mn_20_Ga_20_Gd_20_Co_20_	500 °C	0.5 T	10 mm/s	20%

**Table 2:** The element distribution of the samples by EDS analysis.

Scan Area in SEM Image	Actual Compositions (at.%)					Approximate Atomic Ratio
	Ni	Mn	Ga	Gd	Co	
①	19.3	20.1	20.4	19.8	20.4	1:1:1:1
②	12.4	12.6	12.6	29.8	32.6	1:1:1: 2.4:2.6
③	20.1	20.3	19.8	19.7	20.1	1:1:1:1
④	13.4	13.8	13.7	28.9	30.2	1:1:1:2.1:2.2
⑤	19.8	19.9	20.3	19.8	20.2	1:1:1:1:1
⑥	16.1	16.3	16.2	24.7	26.7	1:1:1:1.5:1.7
⑦	19.9	20.6	19.5	20.5	19.5	1:1:1:1

## Data Availability

The data presented in this study are available on request from the corresponding author.
